# Sub-lattice of Jahn-Teller centers in hexaferrite crystal

**DOI:** 10.1038/s41598-020-63915-7

**Published:** 2020-04-27

**Authors:** V. V. Gudkov, M. N. Sarychev, S. Zherlitsyn, I. V. Zhevstovskikh, N. S. Averkiev, D. A. Vinnik, S. A. Gudkova, R. Niewa, M. Dressel, L. N. Alyabyeva, B. P. Gorshunov, I. B. Bersuker

**Affiliations:** 1 Institute of Physics and Technology, Ural Federal University, Ekaterinburg, Russia; 2 South Ural State University, Chelyabinsk, Russia; 3 Hochfeld-Magnetlabor Dresden (HLD-EMFL), Helmholtz-Zentrum Dresden-Rossendorf, Dresden, Germany; 4 M.N. Miheev Institute of Metal Physics, UB of the RAS, Ekaterinburg, Russia; 5 A.F. Ioffe Physical Technical Institute of the RAS, St. Petersburg, Russia; 6 Moscow Institute of Physics and Technology (State University), Dolgoprudny, Russia; 7 Institute of Inorganic Chemistry, University of Stuttgart, Stuttgart, Germany; 8 1. Physikalisches Institut, Universität Stuttgart, Stuttgart, Germany; 9 Institute for Theoretical Chemistry, the University of Texas at Austin, Austin, TX USA

**Keywords:** Acoustics, Ferroelectrics and multiferroics, Terahertz optics

## Abstract

A novel type of sub-lattice of the Jahn-Teller (JT) centers was arranged in Ti-doped barium hexaferrite BaFe_12_O_19_. In the un-doped crystal all iron ions, sitting in five different crystallographic positions, are Fe^3+^ in the high-spin configuration (S = 5/2) and have a non-degenerate ground state. We show that the electron-donor Ti substitution converts the ions to Fe^2+^ predominantly in tetrahedral coordination, resulting in doubly-degenerate states subject to the $E\otimes e$ problem of the JT effect. The arranged JT complexes, Fe^2+^O_4_, their adiabatic potential energy, non-linear and quantum dynamics, have been studied by means of ultrasound and terahertz-infrared spectroscopies. The JT complexes are sensitive to external stress and applied magnetic field. For that reason, the properties of the doped crystal can be controlled by the amount and state of the JT complexes.

## Introduction

The Jahn-Teller effect (JTE) was predicted more than 80 years ago^1^, and the phenomenon is observed in many branches of natural sciences - physics, chemistry and biology - since it deals with such fundamental property of a system as its symmetry. Originally introduced as breaking off the initial high-symmetry configuration due to the electron-vibrational interaction, it was later extended to slightly distorted states (pseudo-Jahn-Teller effect, see for example^2^, and references therein) and even to other types of coupling (e.g., phonon-strain coupling^3^). Although the JTE results from electron-phonon coupling and is not a spin-dependent phenomenon, it can indirectly affect the spin-dependent parameters via spin-orbit interaction. The systems, where it can be observed, include single crystals^4–7^, thin films^8^, non-organic and organic molecules^9–11^, and even biomolecules^12–15^. The JTE approach is used for description of properties of functional materials, such as semiconductors^16–18^, magnetic materials^5,6,19–21^, superconductors^22^, optical materials^10,18^, and multiferroics^4,17,23,24^. In crystals, the JTE can manifest itself local or uniform. In the first case, the JTE is represented by point Jahn-Teller (JT) centers with considerably low concentration. While, in the second case, it occurs if every elementary cell contains the JT center (see, for example, chapter 8 in^25^); it is called cooperative JTE and leads to a structural phase transition. Frequently the JT centers are $3d$ ions which are responsible for magnetic and transport properties depending on magnetic field. This can lead to possible spintronic applications^26^. Presently, new emerged trends in application of materials manifesting the JTE are a high-precision force sensing^27^ and quantum computing^28,29^.

In crystals, the JT complexes can be artificially produced by irradiation defects or by substitution of a metal ion in cubic, tetrahedral or octahedral coordination by an ion with an orbitally degenerate ground state. Here, we report on a novel and quite different approach to create such complexes that employs specific properties of a certain class of crystals. Though it is also based on doping the crystal with metal ions, there are important differences concerning (i) the properties of the impurity and (ii) the position where it is introduced. An important criterion for choosing the impurity in our approach is its ability to change the charge state of the host metal ion, so that its orbital non-degeneracy is transferred into a degenerate state. This means, that the former requirement on the degeneracy of the impurity ion is eliminated. Another requirement concerning the specific position where the dopant should be inserted is also relaxed; now it may be absolutely arbitrary. It is important, that the JT complex is now represented not by the impurity itself but by the host lattice chemical element whose charge state is changed. As it will be shown below, such approach makes it possible to create a sub-lattice of the JT centers, provided the concentration of the dopant is sufficient. We would like to emphasize the last statement, since the previously known methods produce a subsystem of the JT centers with their random distribution over cation sites but not the sub-lattice structure. This is an important difference, particularly in case of crystals with several magnetic sub-lattices.

**Figure 1:**
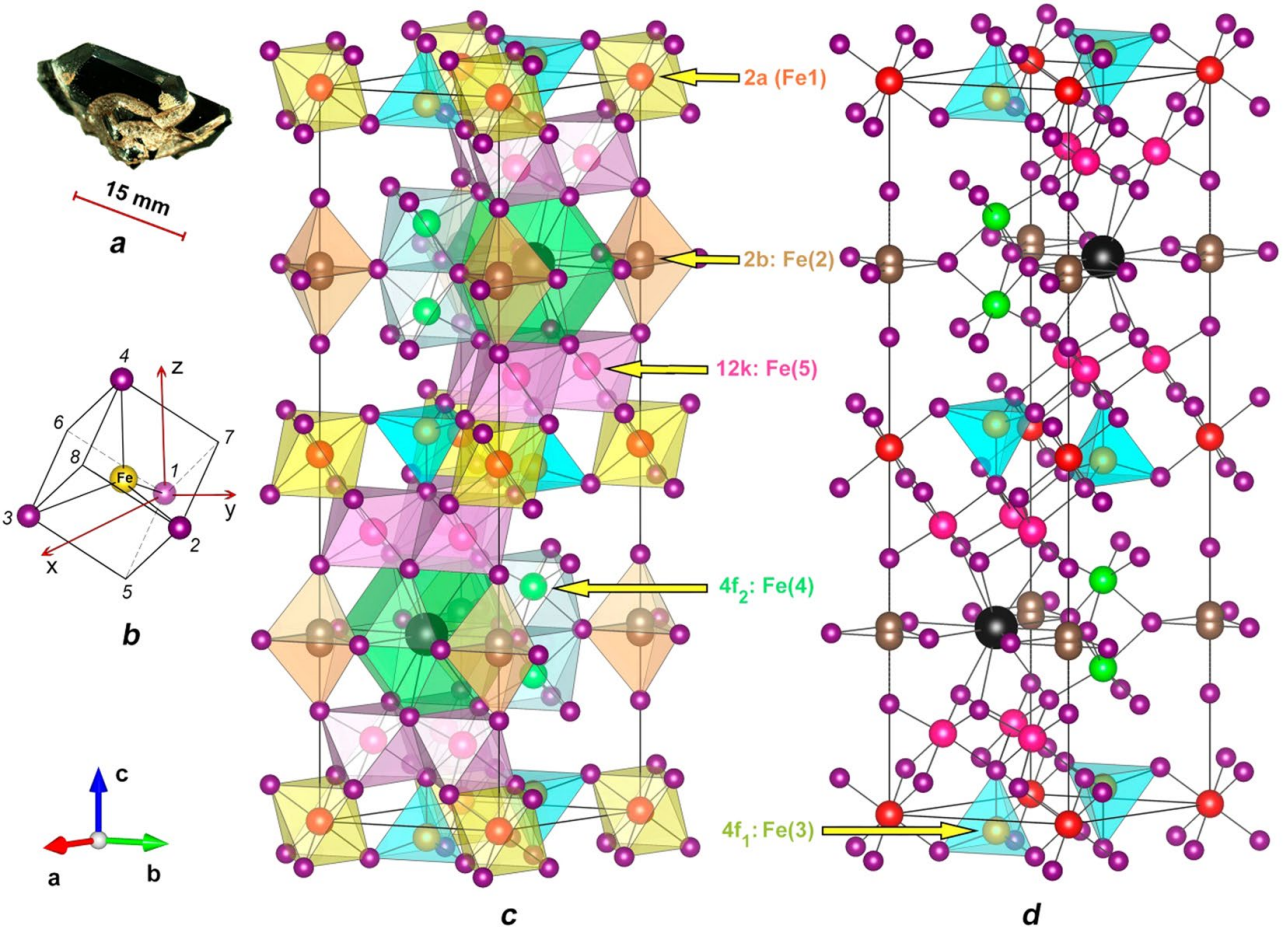
(**a**) Ti-doped hexaferrite BaFe_12_O_19_ single crystal. (**b**) The iron ion Fe(3) in the 4f_1_ tetrahedral coordination in the cube shown in Cartesian system (*z*-axis is parallel to hexagonal *c* -axis; *y*-axis is parallel to *b*-axis). The JT local deformations occur along the edges of the cube 1–5, 1–6 and 1–7; they all are inclined with respect to hexagonal *c-*axis. (**c**) Structure of magnetoplumbite according to^30^: barium is gray (large), oxygen is purple, Fe(1) is red (position 2a), Fe(2) is brown (2b), Fe(3) is olive (4f_1_), Fe(4) is small green (4f_2_), Fe(5) is pink (12k). (**d**) A clarified view of the sub-lattice of the JT centers in tetrahedral coordination.

We have implemented our approach using Ti-doped barium hexaferrite BaFe_12-x_Ti_x_O_19_ ($x=0.75$) crystals. The un-doped material has a crystal structure shown in Fig. 1c (data from ref. ^30^). With the small amount of the dopant (Ti) with respect to the number of the iron ions, we can consider such system as a dilute crystal. In an un-doped crystal, all iron ions have the Fe^3+^ state (free ion has ${d}^{5}$ configuration) and are in the high spin configuration ($S=5/2$) in five different crystallographic positions with octahedral (2a, 4f_2_, 12k in Wyckoff notation), tetrahedral (4f_1_), and bi-pyramidal (2b) coordination (Fig. 1c) in a non-degenerate ${}^{6}A_{1}({t}_{2}^{3}{e}^{2})$ ground state (see Table 1 in page 94 in ref. ^31^). We note, that trigonal bi-pyramidal site-position 2b is known to be unstable and splits into three site-positions, 2b trigonal bi-pyramid itself, where the iron ion is located on the mirror plane of the polyhedron, and two tetrahedra 4e, thus providing additional tetrahedral environment for the iron ions in the lattice. Electron-donor titanium doping leads to change of the charge state of some of the iron ions to Fe^2+^ (${d}^{6}$), converting their former ground state configuration to ${}^{5}{T}_{2}({t}_{2}^{4}{e}^{2})$ in octahedral and ${}^{5}E({t}_{2}^{3}{e}^{3})$ in tetrahedral coordination, both subjected to the JTE (see Table 1 in ref. ^31^). Our experiments confirm that the JT complexes are formed by the iron ions in tetrahedral coordination and the orbital movement of the electrons couples with the local vibrational mode of the tetragonal $e$ symmetry.

**Figure 2:**
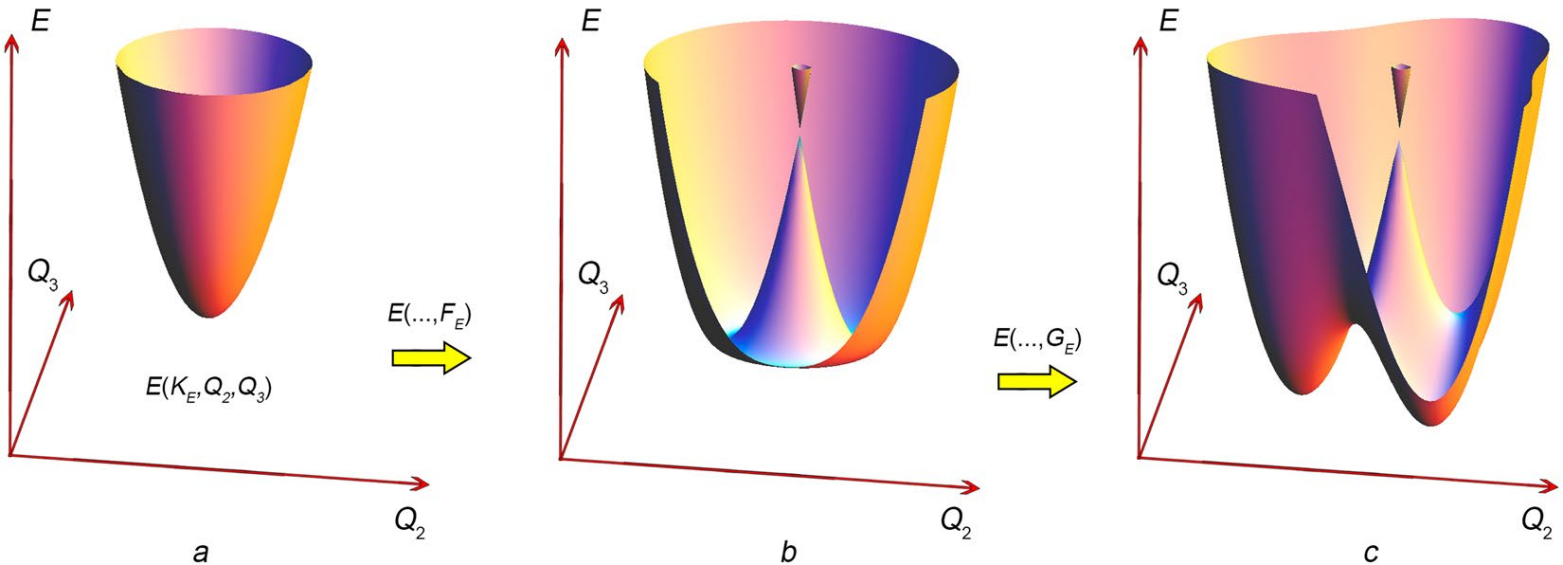
Adiabatic potential energy surface of a tetrahedral Jahn-Teller complex corresponding to the $E\otimes e$ JTE problem: (**a**) without accounting for the vibronic interaction, (**b**) with account of vibronic Hamiltonian taken in linear approximation over $({Q}_{2},{Q}_{3})$, and (**c**) with account of vibronic Hamiltonian in quadratic approximation. In (**a**), the zero potential energy relates to the minimum of $E({Q}_{2},{Q}_{3})$, while the zero energy shown in panels (**b**,**c**) relates to the conical intersection; i.e., account of the vibronic interaction lowers the values of potential energy minima.

For the doubly degenerate *E-*state, the case is referred to as the $E\otimes e$ JTE problem. If the impurity concentration is small (i.e., the distance between the impurities is large), the JT complexes can be considered as non-interacting. The properties of the JT complex are described by the vibronic Hamiltonian (see, for example, ref. ^31^). The calculated adiabatic potential energy surface (APES) is defined in three symmetrized coordinates ${Q}_{i}$ and represents a paraboloid centered at the point of high (4^th^ order) symmetry ${Q}_{2}={Q}_{3}=0$ in the initial state (Fig. 2a). Account of the vibronic interaction in a linear approximation with respect to ${Q}_{i}(i=2,3)$ leads to the APES of the form commonly known as “Mexican hat” (Fig. 2b). In a quadratic approximation, the APES has three minima (Fig. 2c) corresponding to the deformations along the edges of the cube in which the tetrahedron can be inserted (Fig. 1b). As any quantum system with a potential well, it has a discrete set of energy levels called vibronic levels since they correspond to vibrations of a complex described by the symmetry coordinates. Vibrations in the radial direction from the point ${Q}_{2}={Q}_{3}=0$ are characterized by the radial vibronic frequency ${\omega }_{R}$, which is the measure of the energy between the levels in the well. In a non-perturbed (mechanically non-distorted) crystal, the JT complexes are uniformly distributed over the states in equivalent minima and spontaneous transitions occur between the states located in different minima. The transitions can be realized by three mechanisms: thermal activation over the barrier, two-phonon mechanism similar to Raman scattering, and tunneling through the barrier (see, for example, ref. ^31^). At low temperatures, the last one is the most efficient and the JT complexes represent the object with quantum dynamics.

Conventional experimental studies of the JTE are based on optical^32–34^ and spin resonance^35,36^ techniques; ultrasonic technique is used less often. Earlier experiments were done on corundum Al_2_O_3_, yttrium aluminum garnet, and lithium gallium spinel crystals doped with Mn^3+^ and Ni^3+^ ^37^. A detailed description of the experiments carried out by 1967 and their interpretation was given by Sturge^31^. Later the JTE was studied in semiconductors GaAs^38^, ZnSe^39–41^, CdSe^42^, and insulating fluorite SrFe_2_^43^ doped by transition-metal ions. When propagating through the crystal, ultrasonic wave unequally shifts the APES minima and introduces non-equilibrium distribution of the JT complexes over the energy levels. Consequently, the excited state relaxes to the equilibrium with characteristic time $\tau$. The relaxation process introduces an additional channel of the energy loss and, thus, results in the ultrasonic wave attenuation and dispersion (i.e., in nonzero contribution to the imaginary and real parts of the complex wave vector, respectively). Ultrasonic experiments proved to be very useful in investigating the JTE in doped crystals since they provide information on the symmetry properties and APES parameters, and the symmetry of local JT distortions (see, e.g.^44^).

Below we present the results of ultrasonic and terahertz-infrared studies of Ti-doped hexaferrite BaFe_12_O_19_ performed at temperatures 2–200 K in external magnetic field up to 13 T. The analysis is based on a former developed method^43^, modified to be informative with respect to the relaxation mechanisms. The terahertz-infrared spectroscopy allows us to determine the crystallographic sites responsible for the arrangement of the JT complexes.

## Results

### Material

Magnetic oxides, containing iron (“ferrites”), crystallize in different structures. These materials are widely utilized or are promising candidates to be incorporated in various electronic devices owing to their magnetic, insulating, magnetoelectric and multiferroic properties^45–51^. Compounds with the general formula MFe_12_O_19_ (M = Ba, Pb, Sr, Ca, etc.) are prototypes for all hexagonal ferrites. So-called M-type hexaferrites are structurally isomorphic to mineral magnetoplumbite^48^. They exhibit a pronounced magnetocrystalline anisotropy, high Curie temperature (${T}_{C}\approx 750\,{\rm{K}}$), relatively high saturation magnetization ${M}_{s}\approx 72\,{{\rm{A}}{\rm{m}}}^{2}{{\rm{k}}{\rm{g}}}^{-1}$, high coercivity and excellent chemical stability. The structure of un-doped BaFe_12_O_19_ is characterized by space group $P{6}_{3}/mmc$ with the unit cell parameters $a\approx 5.89\,\mathop{{\rm{A}}}\limits^{{\rm{o}}}$ and $c\approx 23.2\,\mathop{{\rm{A}}}\limits^{{\rm{o}}}$^30,52^. An example of single crystal grown for our study is shown in Fig. 1a. The iron ions occupy five different crystallographic positions with octahedral (in 12k, 4f_2,_ and 2a), tetrahedral (in 4f_1_) and trigonal bi-pyramidal (in 2b) coordination, as shown in Fig. 1c. In the centrosymmetric structure, the iron ions in a bi-pyramid occupy the site at the local mirror plane. The Mössbauer and neutron diffraction experiments^53,54^ confirm that at room temperature the iron ions dynamically occupy the 4e positions (in tetrahedrons those constitute the bi-pyramid). Substitution mechanism in the Ba(Fe,Ti)_12_O_19_ compounds depends strongly on the growth conditions. Taking into account the cell parameters dependence on the Ti content^55^ we can conclude that creation of the pairs Ti^4+^/Fe^2+^ is the main mechanism of substitution in BaFe_11.25_Ti_0.75_O_19_ crystals. Although influence of O-deficiency on the Fe^2+^ appearance should not be completely excluded.

### Ultrasonic phase velocity and attenuation

**Figure 3:**
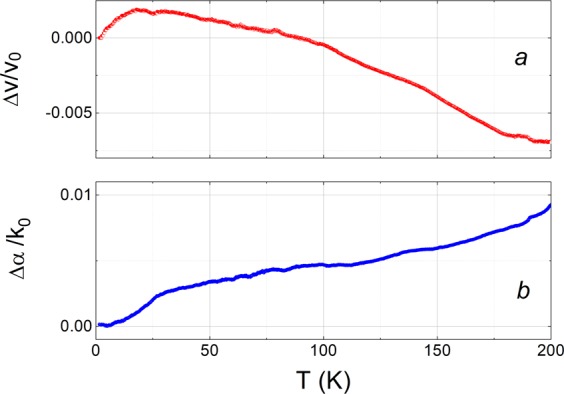
Temperature dependence of the phase velocity (**a**) and attenuation (**b**) of the ultrasonic ${c}_{33}$ longitudinal mode in the BaFe_12-x_Ti_x_O_19_ (x = 0.75) crystal. Wave vector (direction of the wave propagation) ${\bf{k}}//[001]$. ${k}_{0}=\omega /v({T}_{0})$, $\omega /2\pi =23\,{\rm{M}}{\rm{H}}{\rm{z}}$, $\Delta v=v(T)-v({T}_{0})$, $\Delta \alpha =\alpha (T)-\alpha ({T}_{0})$, ${T}_{0}=6\,{\rm{K}}$.

**Figure 4:**
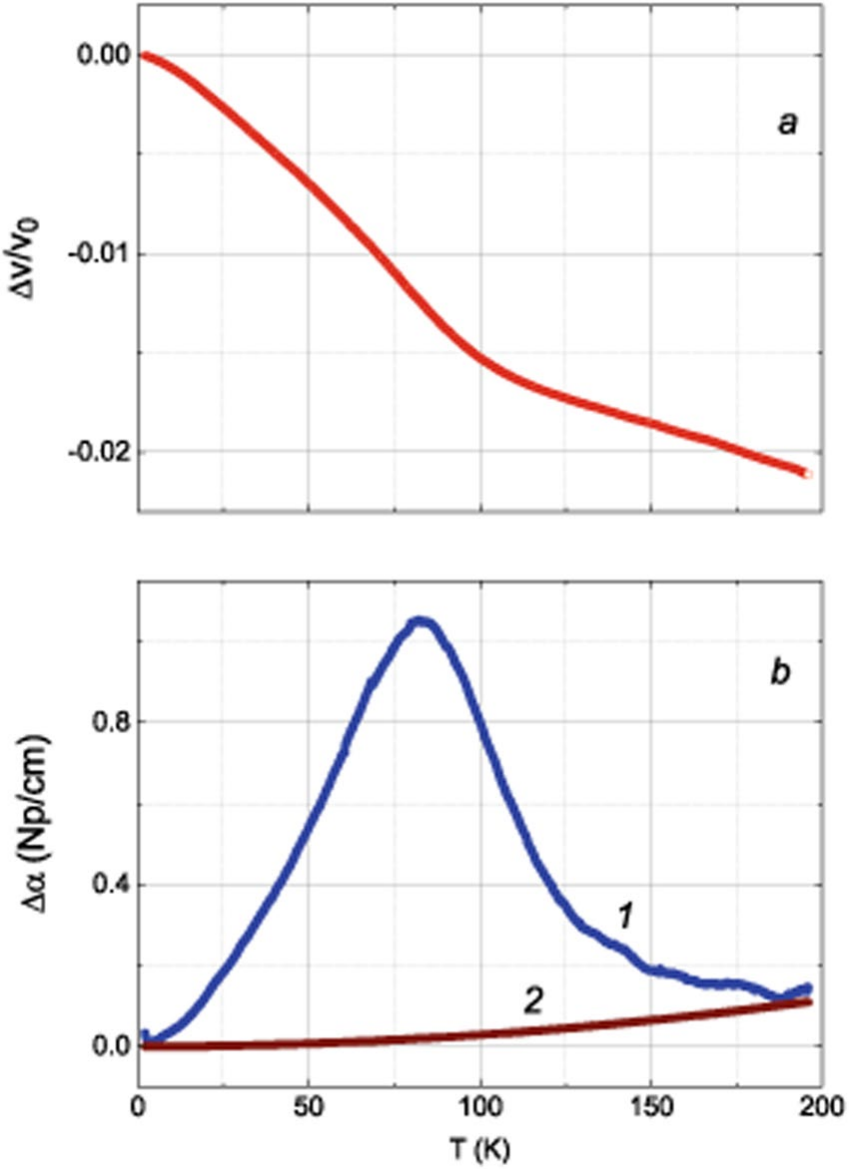
Temperature dependence of the phase velocity (**a**) and attenuation (**b**, curve 1) of the ultrasonic ${c}_{44}$ mode in the BaFe_12-x_Ti_x_O_19_ (x = 0.75) crystal. Wave vector ${\bf{k}}//[001]$. Displacement of volume element produced by the wave (i.e., polarization of the wave) ${\bf{u}}\perp [001]$. ${k}_{0}=\omega /v({T}_{0})$, $\omega /2\pi =15\,{\rm{M}}{\rm{H}}{\rm{z}}$, $\Delta v=v(T)-v({T}_{0})$, $\Delta \alpha =\alpha (T)-\alpha ({T}_{0})$, ${T}_{0}=2\,{\rm{K}}$. Curve 2 represents the background attenuation defined as ${\alpha }_{b}(T)=2.9\cdot {10}^{-6}\cdot {T}^{2}\,{\rm{N}}{\rm{p}}/{\rm{c}}{\rm{m}}$.

We have measured the temperature dependences of velocity and attenuation of the transverse and longitudinal ultrasonic normal modes whose wavevector ${\bf{k}}$ was parallel to the hexagonal crystallographic axis. The ${c}_{33}$ longitudinal mode exhibited monotonic dependences (see Fig. 3), in contrast to the transverse mode. Figure 4 shows anomalous features in temperature dependences of the phase velocity and attenuation of the *c*_44_ mode at $T=70\mbox{--}100\,{\rm{K}}$. For ${\bf{k}}//c$, the attenuation and dispersion are determined by the complex elastic modulus, ${c}_{44}$, no matter what the polarization, **u**, of the transverse mode is (see, for example, ref. ^56^). For the sound wave $u={u}_{0}\exp [i(\omega t-{\bf{k}}\cdot {\bf{r}})]$ we obtain

1 $$k=\frac{\omega }{v}-i\alpha =\frac{\omega }{\sqrt{{c}_{44}/d}},$$

where $\omega$ is the circular frequency of the wave, $v$ is the phase velocity, $\alpha$ is the ultrasonic attenuation coefficient, and $d$ is the density of the crystal. For small deviations of $k$, $v$, and ${c}_{44}$ relative to certain values ${k}_{0}$, ${v}_{0}$, and ${c}_{0}$, respectively, we get

2 $$\frac{\Delta k}{{k}_{0}}=-\frac{\Delta v}{{v}_{0}}-i\frac{\Delta \alpha }{{k}_{0}}=-\,\frac{1}{2}\frac{\Delta {c}_{44}}{{c}_{0}},$$

where ${k}_{0}$ and ${c}_{0}$ are the real parts of the corresponding characteristics.

### Manifestation of the Jahn-Teller effect

**Figure 5:**
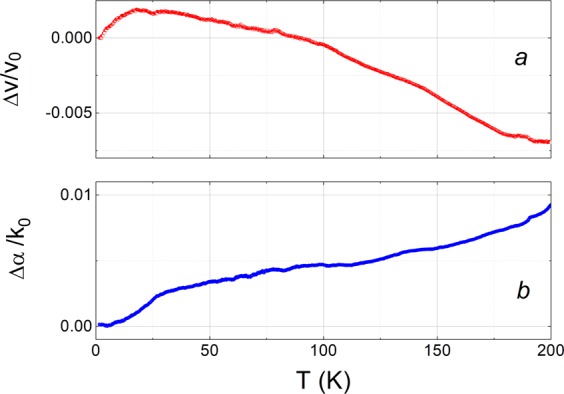
Temperature dependence of the phase velocity (**a**) and attenuation (**b**) of the ultrasonic ${c}_{44}$ mode in the BaFe_12_O_19_ crystal. ${\bf{k}}//[001]$. ${\bf{u}}\perp [001]$. ${k}_{0}=\omega /v({T}_{0})$, $\omega /2\pi =15\,{\rm{M}}{\rm{H}}{\rm{z}}$, $\Delta v=v(T)-v({T}_{0})$, $\Delta \alpha =\alpha (T)-\alpha ({T}_{0})$, ${T}_{0}=1.5\,{\rm{K}}$.

The results presented in Fig. 4 are very similar to those obtained for another hexagonal crystal, CdSe:Cr^2+^ (space group $P{6}_{3}mc$)^42,57^. No anomalies of the attenuation and phase velocity (elastic modulus) have been observed in CdSe:Cr^2+^ for the *c*_33_ mode (longitudinal mode with the wave vector ${\bf{k}}//[001]$) as well. The JT complexes in CdSe:Cr^2+^ are formed by substitution in tetrahedrons of Cd^2+^ with Cr^2+^. Although Cr^2+^ has triply orbital degenerate states in tetrahedral coordination, the global minima of the JT Cr^2+^Se_4_ complex in CdSe matrix proved to possess tetragonal symmetry such as it can be in the complex with doubly orbital degenerate states. The velocity and attenuation of the ${c}_{44}$ ultrasonic mode in nominally pure BaFe_12_O_19_ crystal (see Fig. 5) do not exhibit anomalies like those observed in Ti-doped crystal (given in Fig. 4). Undoubtedly, the tetrahedral complexes in the studied hexaferrite (Fig. 1b) have to manifest the JTE as well.

In the review^31^, Sturge has outlined that the frequency of ultrasonic waves is too low to excite resonance transition between energy levels. Thus, only relaxation-origin anomalies could be registered. Frequency dependence of such anomalies is given in explicit form by, e.g., Eq. (3) [or (23), (24)]. It is completely defined by factor $\omega \tau$ ($\omega$ is frequency of ultrasonic wave, $\tau$ is relaxation time). Peak of attenuation and transformation of the dynamic modulus from quasi-isothermal (high temperature) to quasi-adiabatic (low temperatures) occurs at $\omega \tau \approx 1$. Increase of ultrasonic frequency will result in shift of these anomalies towards high temperatures. This was experimentally confirmed in^39^.

In nominally pure BaFe_12_O_19_ the octahedrons are significantly distorted^30^ and cannot be subjected to the JTE. In contrast, we will consider bi-pyramidal complexes as those responsible for the JTE, in addition to the tetrahedral ones. Formation of the JT complex in a bi-pyramid takes place if Fe^2+^ leaves the centrosymmetric position and appears in tetrahedral coordination. Thus, both possibilities for the JTE are subject to the $E\otimes e$ problem. A more detailed analysis of the JT centers is given below.

### Relaxation time

**Figure 6:**
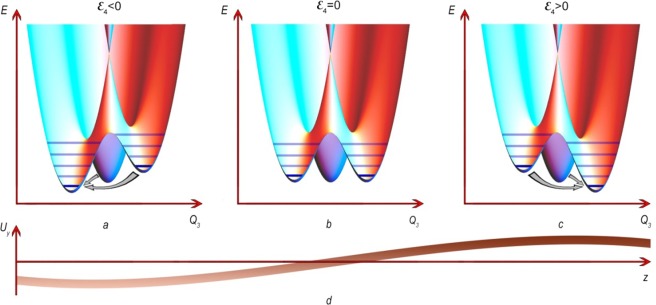
Distortions of the adiabatic potential energy surface that is subject to the $E\otimes e$ problem of the JTE initiated by ultrasonic* c*_44_ mode propagated along the hexagonal axis. The ultrasonic wave produces deformation of the ${\varepsilon }_{4}$ -type, makes the minima nonequivalent and induces non-equilibrium conditions within the JT subsystem. Panels (**a–c**) show the APES distortions due to ${u}_{y}$ displacements in the acoustic wave shown in panel (**d**) at fixed time $t={t}_{0}$. Horizontal lines in (**a–c**) represent the energy levels in the potential wells that are occupied (dark) and unoccupied (light) at low temperatures. One of the energy minima (associated with the edge 1–5 in Fig. 1b) remains unchanged (in the linear approximation over ${\varepsilon }_{4}$) and contains levels that are analogous to those shown in panel (**b**); the energy levels are involved in the relaxation process as shown by gray arrows. This sketch corresponds to a quasi-adiabatic wave propagation with ωτ ≫ 1, i.e., the distribution of JT centers over energy practically does not change. In the opposite case of fast relaxation, ωτ ≪ 1 (quasi-isothermal wave propagation), the relaxation manages to restore the thermal equilibrium much faster than the wave period. In the intermediate case, the non-equilibrium is eliminated only partially.

At first, we calculate the relaxation time using the expression for the dispersion and attenuation of a normal mode, Eq. (2), to identify a contribution of the JT subsystem to the elastic modulus. Since the elastic modulus is the second order derivative of the Helmholtz free energy $F$ or internal energy $U$ with respect to deformation, ${\varepsilon }_{i}$ (see, for example, ref. ^58^), the elastic modulus can also be presented as a sum of contributions related to all subsystems of a crystal, and one of the summands will represent the JT contribution. The energy of phonons used in experiments is too small to induce resonant transitions, implying that the attenuation and dispersion can have only relaxational origin. Ultrasonic wave shifts the minima of the APES and induces non-equilibrium distribution of the JT complexes over the energy states, as shown in Fig. 6. The relaxation tends to establish the equilibrium and is characterized by certain relaxation time $\tau$. The contribution of this process to the dispersion and attenuation of the acoustic wave is

3 $$\frac{\mathrm{Re}{k}_{rel}-i{\alpha }_{rel}}{{k}_{0}}\approx -\,\frac{1}{2}\frac{{c}_{rel}}{{c}_{0}}=\frac{1}{2}\frac{({c}_{JT}^{S}-{c}_{JT}^{T})}{{c}_{0}}\frac{1-i\omega \tau }{1+{(\omega \tau )}^{2}},$$

where the subscripts $rel$ and $JT$ indicate relaxational impact of the JT subsystem, and the superscripts $S$ and $T$ refer to adiabatic and isothermal moduli, respectively. Note, that ${k}_{0}$ and ${c}_{0}$ are related to the whole crystal (not to the JT subsystem). According to^31^, ${c}_{JT}^{S}$ vanishes, while

4 $${({c}_{JT}^{T})}_{ii}=-\,n{k}_{B}T{\left(\frac{{\partial }^{2}\mathrm{ln}Z}{\partial {{\varepsilon }_{i}}^{2}}\right)}_{\varepsilon =0},$$

where $n$ is the concentration of the JT complexes, ${k}_{B}$ is the Boltzmann constant, and the partition function, $Z$, takes into account the shifts of the (populated) energy levels $\Delta {E}_{m}$ produced by the deformations:

5 $$Z=\sum _{m}\exp \left(-\frac{\Delta {E}_{m}}{{k}_{B}T}\right).$$

According to ref. ^59^, the corresponding moduli are:

6 $${({c}_{JT}^{T})}_{33}=0,\,\,{({c}_{JT}^{T})}_{44}={({c}_{JT}^{T})}_{55}=2{({c}_{JT}^{T})}_{11}=2{({c}_{JT}^{T})}_{66}=-\,\frac{1}{18}\frac{n{a}^{2}{F}_{E}^{2}}{{k}_{B}T},$$

where ${F}_{E}$ is the linear vibronic coupling constant and $a$ is the distance between oxygen ions in the tetrahedron. Obviously, information about the ${c}_{33}$ and ${c}_{44}$ moduli is sufficient to describe manifestation of the JTE in BaFe_12-x_Ti_x_O_19_ in an ultrasonic experiment.

7 $$\frac{\mathrm{Re}{k}_{rel}-i{\alpha }_{rel}}{{k}_{0}}=-\,\frac{1}{2}\frac{{({c}_{JT}^{T})}_{44}}{{c}_{0}}\frac{1-i\omega \tau }{1+{(\omega \tau )}^{2}}=\frac{1}{36}\frac{n{a}^{2}{F}_{E}^{2}}{{c}_{0}{k}_{B}T}\frac{1-i\omega \tau }{1+{(\omega \tau )}^{2}}.$$

Extraction of the relaxation time from the experimental data on ultrasonic attenuation requires (i) determination of the relaxation attenuation caused by the JT subsystem ${\alpha }_{rel}(T)=\alpha (T)-{\alpha }_{b}(T)$, [where ${\alpha }_{b}(T)$ is the background attenuation due to other contributions], and (ii) calculation of the temperature dependence of the relaxation time using the following expression (see, e.g., ref. ^44^):

8 $$\,\tau (T)=\frac{1}{\omega }\left\{\frac{{\alpha }_{rel}({T}_{1})\cdot {T}_{1}}{{\alpha }_{rel}(T)\cdot T}\pm {\left[{\left(\frac{{\alpha }_{rel}({T}_{1})\cdot {T}_{1}}{{\alpha }_{rel}(T)\cdot T}\right)}^{2}-1\right]}^{1/2}\right\}.$$

**Figure 7:**
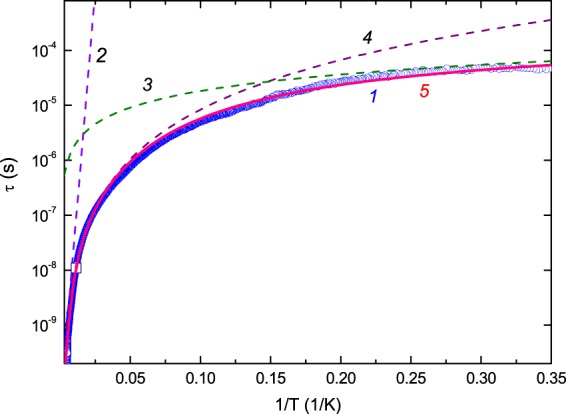
Temperature dependence of the relaxation time in BaFe_12-x_Ti_x_O_19_ (x = 0.75). Square symbol corresponds to $\tau ({T}_{1})$. Blue circles (1) show $\tau$ extracted from the experimental data using Eq. (8). Line 2 corresponds to activated relaxation ${\tau }_{a}=3\cdot {10}^{-11}\exp (700/T)$; line 3 - to tunneling mechanism of relaxation ${\tau }_{t}^{-1}=5.5\times {10}^{3}T$; line 4 - to two-phonon mechanism of relaxation analogous to Raman scattering mechanism ${\tau }_{R}^{-1}=1.5\times {10}^{2}{T}^{3}$. Line 5 (red) represents the sum of all three contributions ${\tau }_{\varSigma }={({\tau }_{a}^{-1}+{\tau }_{t}^{-1}+{\tau }_{R}^{-1})}^{-1}$.

Here ${T}_{1}$ corresponds to the condition $\omega \tau ({T}_{1})=1$. The background attenuation is assumed to be a monotonic function, which coincides with the measured $\Delta \alpha (T)$ at $T\to 0$ and approaches it asymptotically at high temperatures (see Fig. 4b, curve 2). The temperature dependence of the relaxation time from Eq. (8) is shown in Fig. 7.

Sturge^31^ discussed the three mechanisms of relaxation, which we have mentioned above, describing the results of experiments on Al_2_O_3_:Ni^3+^, whose APES had three minima separated by energy barriers. To remind, these mechanisms are thermal activation over the barrier, two-phonon mechanism and tunnelling through the energy barrier. Corresponding relaxation rates are^31^:

9 $${\tau }_{a}^{-1}={\tau }_{0}^{-1}\exp (-{V}_{0}/T),$$

10 $${\tau }_{R}^{-1}=(B/{\theta }^{2}){T}^{3},$$

11 $${\tau }_{t}^{-1}=BT.$$

The relaxation time with these mechanisms taken into account (curve 5 in Fig. 7) is in perfect agreement with the relaxation time extracted from our experiments (curve 1). This evedences the JTE origin of the observed anomalies. Note, that the authors of ref. ^60^ reported the temperature dependences of the Young’s modulus (similar to ${c}_{11}$) and acoustic losses measured at frequencies between 100 and 200 kHz in similar material BaFe_12-x_Ti_x_O_19_ ($x=1$). They related the anomalies observed at around 100 K to thermal activation and estimated the activation energy, ${V}_{0}$, of about 25 meV which can be considered to be in satisfactory agreement with our result, ${V}_{0}=700\,{\rm{K}}=487\,{{\rm{cm}}}^{-1}=60\,{\rm{meV}}=96.6\cdot {10}^{-22}\,{\rm{J}}$, keeping in mind that the anomaly of Young’s modulus in ref. ^60^ was rather small (and the accuracy was low) due to low ultrasound frequency.

### Location of the Jahn-Teller centers

As mentioned above, the iron ions occupy five different crystallographic positions with octahedral (in 12k, 4f_2,_ and 2a), tetrahedral (in 4f_1_) and trigonal bi-pyramidal (in 2b) coordination (see Fig. 1c). All of them are subject to the JTE. We assume that establishing the JT centers caused by charge transfer starts from formation of the complexes with the lowest potential energy (either tetrahedral, bi-pyramidal, or octahedral ones). Once such complexes are composed, further increase of the Ti ions content results in the complexes with higher potential energy, and so on.

The octahedral complexes should not be responsible for the observed anomalies due to the following reasons. (i) They are strongly distorted even in nominally pure BaFe_12_O_19_^30^. (ii) The octahedral coordination of Fe^2+^ leads to triply degenerate ${}^{5}{T}_{2}({t}_{2}^{4}{e}^{2})$ ground state. Consequently, the global minima of the APES can have tetragonal, trigonal or orthorhombic symmetry. The octahedrons are inclined with respect to the hexagonal axis. In such a geometry, there is no case when either all the edges, or all the diagonals or all the face diagonals of the cube in which the octahedron is inserted will be equally elongated (shortened) by longitudinal wave propagating along [0001] axis (the ${c}_{33}$ mode). One or two of them will always differ from the others. It means that the energy levels corresponding to tetragonal, trigonal, or orthorhombic distortions will not be shifted equally by the mentioned longitudinal mode. In such case, the dispersion of the c_33_ mode and attenuation would be of relaxational origin that is not observed in our experiment. The observed combination of relaxational anomalies in attenuation and velocity of the c_44_ mode and their absence in the c_33_ mode, according to ref. ^59^, is indicative of tetragonal distortions of tetrahedral complexes in a hexagonal crystal. So, the octahedral complexes should not be responsible for the observed anomalies. Bi-pyramids constitute the JT complexes if the iron ion leaves the centrosymmetric position and appears in tetrahedral coordination. Such transformation manifests itself in the temperature dependences of material tensors that describe thermodynamic properties of the material. In view of this, we have studied the temperature dependences of the dielectric permittivity and elastic modulus.

### Dielectric permittivity

**Figure 8:**
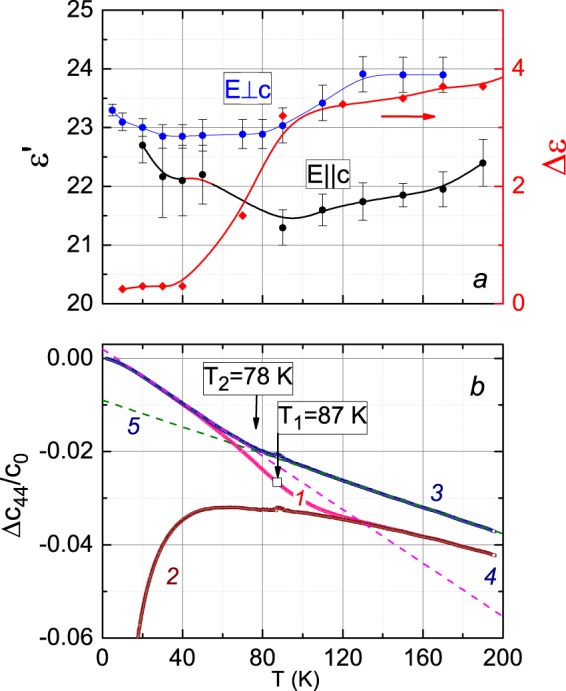
Temperature dependences of dielectric characteristics (**a**) and elastic modulus ${c}_{44}$ (**b**) of single-crystalline BaFe_11.25_Ti_0.75_O_19_. Dielectric permittivity ε′ was measured at the frequency of 0.3 THz (wavenumber 10 cm^−1^) for two principle polarizations of the electric field vector of the probing radiation relative to the c-axis, **E**⊥*c* (blue color) and **E** | | *c* (black color), as indicated. The red dots show the dielectric contribution Δε of the terahertz absorption resonance in BaFe_10.85_Ti_1.15_O_19_. Lines are guides to the eye. The modulus ${c}_{44}$ is presented for three conditions: dynamic (line 1), relaxed (2), and unrelaxed (3). Line 4 corresponds to $\Delta {c}_{44}/{c}_{0}=-\,0.009-0.000143\cdot T$, line 5 - to $\Delta {c}_{44}/{c}_{0}=0.002-0.000287\cdot T$. Here $\Delta {c}_{44}={c}_{44}(T)-{c}_{0}$, ${c}_{0}={c}_{44}({T}_{0})$ and ${T}_{0}=2\,{\rm{K}}$.

We measured the terahertz spectra of complex dielectric permittivity ${\varepsilon }^{\ast }={\varepsilon }^{{\rm{{\prime} }}}+i{\varepsilon }^{{\rm{{\prime} }}{\rm{{\prime} }}}$ of several BaFe_12-x_Ti_x_O_19_ crystals with $x=0.62,\,0.75,\,1.15$ (this work and^61^). The terahertz spectra of Ti- or Pb-substituted compounds were found to be determined mainly by electronic transitions of the tetrahedrally coordinated Fe^2+^ ions (for details see^51^). According to the charge compensation mechanism, in Ti^4+^-substituted BaFe_12-x_ Ti_x_O_19_ hexaferrites with $x < 0.8$, part of the trivalent iron ions restores to the divalent state to maintain charge neutrality at aliovalent substitution. X-ray diffraction and magnetic measurements^55,62^ indicate that Ti ions prefer to occupy octahedral positions instead of tetrahedral ones. Similar indications are provided by neutron diffraction on co-substituted BaFe_12–2x_Ti_x_Co_x_O_19_^63^. The 2b site-position with iron ions situated on a mirror plane in a trigonal bipyramid is known to be subject to splitting into two sub-positions 4e with iron ions located in tetrahedrons. At high enough temperatures, the iron dynamically oscillates between the two states, by cooling, it freezes in one of the states^49^. The critical temperature that corresponds to freezing out of the dynamical oscillation was determined in pure BaFe_12_O_19_ to be around 80 K. At about the same temperatures, clear anomalies were observed in the dielectric characteristics related to the terahertz excitations in BaFe_12-x_Ti_x_O_19_ (Fig. 8a).

Thermal-dispersive behavior observed in the ${c}_{44}$ at *T* = 70–100 K is determined by the background elastic modulus which can be assumed as an adiabatic one (see curve 3 in Fig. 8) and the relaxation-nature contribution of the JT sub-system. The last is described by the Eq. (3) which contains the temperature dependent variables: isothermal modulus ${({c}_{JT}^{T})}_{44}$ given by Eq. (6) and relaxation time $\tau$ shown in Fig. 7. The factor $-1/[1+{(\omega \tau )}^{2}]$ (see, e.g., Fig. 1 in^64^) which enters the relaxation-nature contribution, increases from (−1) in the vicinity of $T={T}_{1}$ (remind, this temperature corresponds to the condition $\omega \tau ({T}_{1})=1$) and vanishes at low temperatures (*T* ≪ *T*_1_).

Anomalous behaviors of the dielectric permittivity at around $80{\rm{K}}$ (Fig. 8a) and change in the slope of the unrelaxed (adiabatic) modulus (line 3 in Fig. 8b) at ${T}_{2}=78\,{\rm{K}}$ reflect a transformation associated with the position of the iron ions in bi-pyramids.

### Dynamic, relaxed, and unrelaxed moduli

We use the term *dynamic modulus* for the measured frequency-dependent modulus in contrast to the frequency independent isothermal ${c}^{T}$ and adiabatic ${c}^{S}$ moduli. Solution of the Zener equation^65^, by keeping only the first derivatives over time, gives expressions for the dynamic modulus ${c}_{d}$^66^ [Eqs. (22) or (23)]. Note, Zener used the terms *relaxed* and *unrelaxed* for isothermal and adiabatic moduli, respectively. It should be emphasized, that these equations are obtained for the case with only one relaxation process in which the whole system is involved. In general, the measured ${c}_{d}$ can include relaxation or resonance contributions from other than the JT subsystem (non-JT impurities, vacancies, or crystal imperfections). However, the subsystem of JT complexes is very special: due to the well-defined multi-minimum APES, strong anisotropy and sensitivity to the polarization of ultrasound wave, it produces specific anomalies, seen by ultrasound absorbers. Ultrasonic experiments can provide information not only about the *dynamic* modulus ${c}_{d}$ [Eq. (21)] but about the *adiabatic* (unrelaxed) modulus ${c}^{U}$ of the whole system [Eq. (26)] and the modulus ${c}^{R}$ [Eq. (27)] which we call *relaxed* one. The last contains contribution of the background modulus (which can be adiabatic) and isothermal contribution of the JT subsystem. Note, such definition differs from one introduced by Zener.

The temperature dependences of the dynamic, relaxed and unrelaxed moduli are displayed in Fig. 8b. With temperature increase, one can see a typical for the JTE transformation of $\mathrm{Re}{c}_{d}$ from the unrelaxed to the relaxed regime in the vicinity of ${T}_{1}=87\,{\rm{K}}$. Besides, the unrelaxed modulus exhibits a change in slope at ${T}_{2}=78\,{\rm{K}}$(Fig. 8b). This anomaly can be attributed to the same transformation mentioned above. However, the anomaly caused by the JTE is more pronounced; it is located close to ${T}_{2}$ and, thus, masks the change of the slope in $\mathrm{Re}{c}_{d}(T)$. The method of extracting the unrelaxed modulus based on analysis of the ultrasonic phase velocity and attenuation, proves to be very useful. Keeping in mind that the JTE caused by bi-pyramids is expected to be below $78\,{\rm{K}}$, we have to *exclude* these complexes as being responsible for the JTE seen at higher temperatures (see Fig. 4). There are no signs of an overlap of the two contributions. We, thus, conclude that the iron ions Fe(3) in tetrahedral positions 4f_1_ are responsible for the JTE.

### Ground state and adiabatic potential energy surface

In the framework of the $E\otimes e$ problem of the JTE, the APES is a function of tetragonal symmetrized coordinates $({Q}_{2},{Q}_{3})$. In quadratic approximation in polar coordinates $(\rho ,\phi )$ (see, e.g., p.53–54 in ref. ^25^) it has a form

12 $${\varepsilon }_{\pm }^{\nu }(\rho ,\phi )=\frac{1}{2}{K}_{E}{\rho }^{2}\pm \rho {[{F}_{E}^{2}+{G}_{E}^{2}{\rho }^{2}+2{F}_{E}{G}_{E}\rho \cos (3\phi )]}^{1/2},$$

where ${\rho }^{2}={Q}_{2}^{2}+{Q}_{3}^{2}$, ${K}_{E}$ is the primary force constant (defined without account of vibronic interaction), ${F}_{E}$ and ${G}_{E}$ are the linear and quadratic coupling constants, respectively. Since the temperature dependence of the relaxation time indicates activation regime at $T > {T}_{1}$ and has the tendency towards saturation at low temperatures, we conclude that the APES has energy barriers [see Fig. 2c] and ${G}_{E}\ne 0$. In this case, the extremal points are located at

13 $${\rho }_{q}={\frac{\pm {F}_{E}}{{K}_{E}\mp {(-1)}^{n}2{G}_{E}}}_{0}=\frac{{\rho }_{0}}{1\mp [{(-1)}^{n}2{G}_{E}/{K}_{E}]},\,{\phi }_{0}=n\pi /3,\,{\rm{n}}=0,1,\mathrm{..}.5,$$

where ${\rho }_{0}=\pm \,{F}_{E}/{K}_{e}$. The upper and lower signs correspond to ${F}_{E} > 0$ and ${F}_{E} < 0$, respectively. If ${F}_{E}{G}_{E} > 0$, the points with $n=0,2,4$ are minima, and those with $n=1,3,5$ are saddle points, whereas for ${F}_{E}{G}_{E} < 0$ these two types of extrema interchange. For the JT stabilization energy ${E}_{JT}$ we have

14 $${E}_{JT}=\frac{{F}_{E}^{2}}{2({K}_{E}-2|{G}_{E}|)}$$

and the minimal barrier height $\delta$ between the minima is

15 $$\delta =\frac{4{E}_{JT}|{G}_{E}|}{({K}_{E}+2|{G}_{E}|)}.$$

The expression for the barrier height can be introduced in terms of the activation energy [entering Eq. (9) and obtained from experimental data ${V}_{0}=700\,{\rm{K}}=96.6\cdot {10}^{-22}\,{\rm{J}}$] and the energy of zero vibrations

16 $$\delta ={V}_{0}+\frac{1}{2}\hslash {\omega }_{R},$$

where ${\omega }_{R}$ is the radial vibronic frequency which can be taken as equal to the frequency of the shear acoustic phonon mode ${\omega }_{L1}$ ($\hslash {\omega }_{L1}=92.45\,{{\rm{cm}}}^{-1}=1.834\cdot {10}^{-21}\,{\rm{J}}$^50^). Consequently, $\delta =533\,{{\rm{cm}}}^{-1}=1.06\cdot {10}^{-20}\,{\rm{J}}$.

Equation (15) represents a quadratic equation with respect to $|{G}_{E}|$ with a positive root:

17 $$|{G}_{E}|=\left(-\frac{{F}_{E}^{2}}{2\delta }+\sqrt{\frac{{F}_{E}^{4}}{4{\delta }^{2}}+\frac{{K}_{E}^{2}}{4}}\right).$$

The linear constant of vibronic coupling ${F}_{E}$ can be derived from the data on ultrasonic attenuation. We have to rewrite Eq. (7) for $T={T}_{1}$, allocate the real part and solve it with respect to ${F}_{E}^{2}$:

18 $${F}_{E}^{2}=72\frac{{\alpha }_{rel}({T}_{1}){c}_{0}{k}_{B}{T}_{1}}{n{a}^{2}{k}_{0}}.$$

The Fe^2+^ ions in tetrahedrons originate from the Ti doping. For BaFe_12-x_Ti_x_O_19_ with x = 0.75, this gives ${n}_{{\rm{Ti}}}=2.1\cdot {10}^{25}\,{{\rm{m}}}^{-3}$. We assume the concentration of the JT centers as $n={n}_{{\rm{Ti}}}$ that corresponds to the low-limit estimates of ${F}_{E}^{2}$ and ${E}_{JT}$(the validity of this assumption is discussed below). Then, we get $|{F}_{E}|=2.1\cdot {10}^{-9}\,{\rm{N}}$. In addition to ${n}_{{\rm{Ti}}}$ and $a=3.126\,\mathop{{\rm{A}}}\limits^{{\rm{o}}}$, we have used the following values: ${c}_{0}={c}_{44}({T}_{0})=37.6\cdot {10}^{9}\,{{\rm{J}}/{\rm{m}}}^{3}$, ${k}_{0}=3.5\cdot {10}^{4}\,{{\rm{m}}}^{-1}$, ${\alpha }_{rel}({T}_{1})\cdot {T}_{1}=8.73\cdot {10}^{3}\,{\rm{K}}\cdot {\rm{Np}}/{\rm{m}}$. With the values of the linear vibronic coupling constant and the primary force constant ${K}_{E}=94\,{{\rm{J}}/{\rm{m}}}^{2}$ (see Section Methods), we get ${E}_{JT}=1.5\cdot {10}^{3}\,{{\rm{cm}}}^{-1}=3.0\cdot {10}^{-20}\,{\rm{J}}$, $|{G}_{E}|=9.9\,{{\rm{J}}/{\rm{m}}}^{2}$, and ${\rho }_{0}=4.8\cdot {10}^{-11}\,{\rm{cm}}$.

### Magnetic field dependence of ultrasonic attenuation

**Figure 9:**
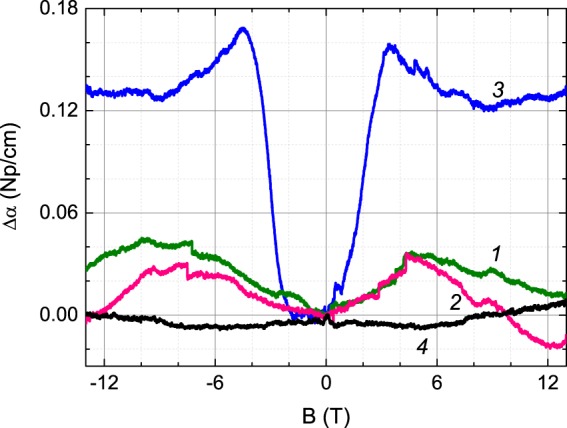
Magnetic field dependence of the attenuation of the ultrasonic ${c}_{44}$ mode obtained at $T=2\,{\rm{K}}$ (line 1), $20\,{\rm{K}}$ (2), $80\,{\rm{K}}$ (3), and $145\,{\rm{K}}$ (4). The experimental geometry is ${\bf{B}}$//${\bf{k}}//[001]$, ${\bf{u}}\perp [001]$. The ultrasound frequency is $\omega /2\pi =15\,{\rm{MHz}}$. The measurements were performed with sweeps from the highest positive to the negative field. Shift of the symmetry plane towards negative $B$ values by approximately −1 T is due to the magnetic hysteresis.

To find out how the magnetic field influences the state of the JT centers, we have performed the measurements of phase velocity and attenuation of the acoustic ${c}_{44}$ mode as function of magnetic field. While both ultrasonic attenuation and velocity are influenced by magnetic field, a more pronounced contribution from the JT subsystem was observed for the attenuation (see Fig. 9). Obviously, the anomaly observed for the $80{\rm{K}}$ data has JT origin and correlates with the attenuation maximum in Fig. 4b.

Expanding the expression for dispersion and attenuation [Eq. (3)] to the case of non-zero magnetic field requires (i) substitution of ${c}^{S}$ and ${c}^{T}$ by ${c}^{S,B}$ and ${c}^{T,B}$, respectively, and (ii) considering the magnetic field dependence of the relaxation time: $\tau =\tau (T,B)$^66^. It was shown^40^ that magnetic field provided a new channel of relaxation. This increases the total relaxation rate according to

19 $$\frac{1}{\tau (T,B)}=\frac{1}{{\tau }_{T}}+\frac{1}{{\tau }_{B}},$$

where ${\tau }_{T}$ is the temperature dependent relaxation time at $B=0$ and ${\tau }_{B}$ accounts for the magnetic field contribution at fixed temperature. At high temperatures, $\omega {\tau }_{T}\ll 1$, $\alpha /{k}_{0}\propto (\omega \tau )$, the relaxation is fast and the contribution from the magnetic field is negligible. At low temperatures, $\omega {\tau }_{T}\gg 1$, $\alpha /{k}_{0}\propto {(\omega \tau )}^{-1}$ is a small value. For $\omega \tau \approx 1$_,_ the attenuation is close to its peak value and the magnetic field enhances the relaxation rate, that is qualitatively equivalent to the effect of the temperature increase. Hence, application of a magnetic field *B* at $T=80{\rm{K}}$ should result in an increase of attenuation that is observed for $B\le 4.5{\rm{T}}$. Further increase of magnetic field results in further changes that can be due to the magnetic field dependence of ${c}^{T,B}$. In any case, the low-field attenuation presented in Fig. 9 is as anticipated.

## Discussion and conclusions

The BaFe_11.25_Ti_0.75_O_19_ crystal studied here can be regarded as a diluted magnetic crystal: The given amount of the Ti dopant ions can produce less than 38% of possible JT centers in 4f_1_ sites. These centers can be considered as non-interacting due to a large space separation. On the other hand, iron ions are involved in a cooperative effect that produces magnetic ordering^67^, i.e., magnetically they are strongly coupled with each other. This brings us to an exceptional situation: from the viewpoint of vibronic coupling, we have a system of non-interacting complexes, but from the magnetic point of view, the system is strongly correlated. By investigating such a specific material, we conclude that: (i) the JT complexes in BaFe_11.25_Ti_0.75_O_19_ (JTE $E\otimes e$ problem) are constructed by the charge compensated iron ions in tetrahedral coordination producing a sublattice of the JT centers in 4f_1_ sites (see Fig. 2d), (ii) the JTE in BaFe_11.25_Ti_0.75_O_19_ leads to anomalies in temperature and magnetic field dependences of the ultrasonic attenuation and phase velocity, (iii) the description of the vibronic interaction should be done in the quadratic approximation resulting in the adiabatic potential energy surface with three minima and three saddle points in the ground state, and (iv) the relaxation time is determined by three mechanisms - thermal activation over potential energy barrier, two-phonon mechanism and tunneling through the potential energy barrier. In addition, our analysis of the experimental data allowed us to determine the JT stabilization energy, the linear and the quadratic constants of the vibronic coupling, and the potential energy barrier.

Temperature dependences of the relaxed and unrelaxed elastic moduli ${c}_{44}$ are obtained from the experimental dynamic modulus and the relaxation time. The unrelaxed (adiabatic) modulus (curve 3 in Fig. 8b) confirms the transformation of bi-pyramids at $78\,{\rm{K}}$ that is indicated by our terahertz spectroscopic (Fig. 8a) and earlier optical experiments^50,61^.

The Fe^3+^ ions in 4f_1_ sites have opposite (anti-parallel) spin orientation with respect to the total magnetization. The transformation to divalent state results in local symmetry breaking and leads to emergence of a local selected direction (not parallel to the hexagonal *c*-axis) and cancelling collinearity of the magnetic sub-lattices of the crystal. BaFe_12-x_Ti_x_O_19_ represents a specific type of crystals with the impurity ions (Ti^4+^) randomly distributed over the octahedral and bi-pyramidal sites, but the Fe^2+^ ions appear only in the 4f_1_ positions, provided that amount of the dopand is small ($x\le 2$). At $x=2$ the Fe^2+^ ions occupy all 4f_1_ sites and the crystal should acquire a completed sub-lattice of the JT centers. This artificially constructed sub-lattice of the JT centers is sensitive to external stress and magnetic field giving an opportunity to control the properties of the crystal by the impurity amount, stress and magnetic field.

## Methods

### Sample growth and characterization

Single crystal of the M-type hexaferrite BaFe_12-x_Ti_x_O_19_ ($x=0.75$) was grown by a flux melt technique in a platinum crucible using a high temperature furnace^68^ at the South Ural State University, Russia. As a starting material, the primary reagents of BaCO_3_ and Fe_2_O_3_ were taken with the respective molar percentage (according to the composition). The crystal composition was determined using a scanning electron microscope Jeol JSM7001F equipped with an energy dispersive X-ray fluorescence spectrometer Oxford INCA X-max 80 for elemental analysis. At least seven crystal areas were measured to get the average composition data. The mean deviation for cation content was up to 5%. Crystal shape is prismatic with an irregular hexagonal base of diameter ∼10 mm perpendicular to the easy direction and of height  5 mm. After cutting and polishing the faces for ultrasound transducers the sample had dimension of 4.14  mm (sample length for the propagation of the ultrasonic waves) along the [001] crystallographic axis.

### Ultrasonic technique

Measurements of the ultrasonic phase velocity and attenuation were done at $15\,{\rm{MHz}}$ (transverse waves) and $23\,{\rm{MHz}}$(longitudinal waves) in temperature range $2\mbox{--}200\,{\rm{K}}$ and in magnetic fields up to $13\,{\rm{T}}$. The waves were generated and detected by resonant LiNbO_3_ piezoelectric transducers. The direction of wave propagation (wavevector ${\bf{k}}$) and the applied magnetic field were parallel to the hexagonal crystallographic *c*-axis providing information on the ${c}_{44}$ and ${c}_{33}$ elastic moduli. The experiments were done at High Magnetic Field Laboratory (HLD-EMFL), Helmholtz-Zentrum Dresden-Rossendorf, Germany, and at the Institute of Physics and Technology, Ural Federal University, Russia, using setups operating as a frequency variable bridge. The detailed description can be found in^68,69^. The measurements are based on a pulse-echo method and phase-sensitive detection techniques. High-frequency mechanical oscillations in the piezoelectric transducer were excited by a radio pulse with duration of about $1\,\mu {\rm{s}}$. After propagation through the sample, the oscillations were transformed into an electric signal by another piezoelectric transducer located at the opposite side of the sample. Detected radio pulses were amplified and mixed with two phase-shifted reference signals of the same frequency. After suppressing the second harmonic with low-pass filters and integrating the resulting signals with gated box-car averagers, the output *dc* signal, was used for the frequency modulation, keeping the phase constant and providing information about the sound-velocity changes. Another *dc* signal is related to the ultrasound attenuation. The setup at the Ural Federal University has square-wave frequency and amplitude modulation and two phase detectors operating at modulation frequency ($100\,{\rm{k}}{\rm{H}}{\rm{z}}$). The output of the detectors were used as feedback signal for frequency bridge balance and for detecting the amplitude variation caused by influence of the external parameter (temperature or magnetic field). Details can be found in ref. ^43^. Both setups maintain the fixed phase regime. In this case, the variation of frequency $\varDelta \omega$ required for keeping the phase balance of the bridge, the phase velocity $\Delta v$, and the sample length $\Delta \ell$ variations caused by the external parameters) and their reference magnitudes, ${\omega }_{0}$, ${v}_{0}$, and ${\ell }_{0}$ enter the following expression

20 $$\frac{\Delta \omega }{{\omega }_{0}}=\frac{\Delta v}{{v}_{0}}-\frac{\Delta \ell }{{\ell }_{0}}.$$

Using the relation between the dynamic elastic modulus and phase velocity ${c}_{d}={\rho }_{m}{v}^{2}$ (${\rho }_{m}$ is the mass density) we obtain the following equation for small changes of $v,\,{c}_{d},\,\omega$, and $\ell$:

21 $$\frac{\Delta v}{{v}_{0}}=\frac{1}{2}\frac{\Delta {c}_{d}}{{c}_{0}}=\frac{\Delta \omega }{{\omega }_{0}}+\frac{\Delta \ell }{{\ell }_{0}},$$

Variation of the attenuation coefficient $\Delta \alpha$ introduced as variation of the imaginary part of the complex wave number $k=\mathrm{Re}k-i\alpha$ is given (in neper per cm) as

22 $$\Delta \alpha =-\,\frac{1}{\ell }\,{\rm{l}}{\rm{n}}\,\frac{u}{{u}_{0}},$$

where $u$ is the voltage at the input of the receiver and ${u}_{0}$ is its reference magnitude.

Solution of the Zener equation, keeping only the first derivatives over time, gives the following expressions for the dynamic modulus ${c}_{d}$^65^

23 $${c}_{d}={c}^{S}-({c}^{S}-{c}^{T})\frac{1-i\omega \tau }{1+{(\omega \tau )}^{2}},$$

24 $${c}_{d}={c}^{T}+({c}^{S}-{c}^{T})\frac{{(\omega \tau )}^{2}-i\omega \tau }{1+{(\omega \tau )}^{2}}.$$

Considering the JT subsystem we can express its contribution to the phase velocity in terms of relaxational attenuation and relaxation time using Eqs. (3) and (6):

25 $$\begin{array}{rcl}\frac{{v}_{rel}}{{v}_{0}} & = & \frac{1}{2}\mathrm{Re}\frac{{c}_{rel}}{{c}_{0}}=-\frac{1}{2}\frac{({c}_{JT}^{S}-{c}_{JT}^{T})}{{c}_{0}}\frac{1}{1+{(\omega \tau )}^{2}}=2\frac{{v}_{rel}({T}_{1})\cdot {T}_{1}}{{v}_{0}T}\frac{1}{1+{(\omega \tau )}^{2}}\\  & = & -2\frac{{\alpha }_{rel}({T}_{1})\cdot {T}_{1}}{{k}_{0}T}\frac{1}{1+{(\omega \tau )}^{2}}=-\,\frac{{\alpha }_{rel}}{{k}_{0}}\frac{1}{\omega \tau }.\end{array}$$

Expression (23) makes it possible to calculate the temperature dependence of the unrelaxed ${c}^{U}$ elastic modulus (that contains the background contribution and the adiabatic contribution of the JT subsystem). Provided the background elastic modulus is adiabatic, the unrelaxed modulus represents the adiabatic modulus ${c}^{S}$ of the whole crystal. The relaxed modulus ${c}^{R}$ represents the background contribution and isothermal contribution of the JT subsystem. The derivation of the expressions for ${c}^{U}$ and ${c}^{R}$ given in ref. ^64^ leads to:

26 $$\begin{array}{rcl}\frac{\Delta {c}^{U}}{{c}_{0}} & = & \mathrm{Re}\left[\frac{{c}_{rel}}{{c}_{0}}+\frac{\Delta {c}_{b}}{{c}_{0}}+\frac{({c}_{JT}^{S}-{c}_{JT}^{T})}{{c}_{0}}\frac{1-i\omega \tau }{1+{(\omega \tau )}^{2}}\right]\\  & = & 2\left(\frac{\Delta v}{{v}_{0}}-\frac{{v}_{rel}}{{v}_{0}}\right)=2\left(\frac{\Delta v}{{v}_{0}}+\frac{{\alpha }_{rel}}{{k}_{0}}\frac{1}{\omega \tau }\right),\end{array}$$

27 $$\begin{array}{rcl}\frac{\Delta {c}^{R}}{{c}_{0}} & = & \mathrm{Re}\left[\frac{{c}_{rel}}{{c}_{0}}+\frac{\Delta {c}_{b}}{{c}_{0}}-\frac{({c}_{JT}^{S}-{c}_{JT}^{T})}{{c}_{0}}\frac{{(\omega \tau )}^{2}-i\omega \tau }{1+{(\omega \tau )}^{2}}\right]\\  & = & 2\left(\frac{\Delta v}{{v}_{0}}+\frac{{v}_{rel}}{{v}_{0}}{(\omega \tau )}^{2}\right)=2\left(\frac{\Delta v}{{v}_{0}}+\frac{{\alpha }_{rel}}{{k}_{0}}\omega \tau \right),\end{array}$$

where $\Delta {c}^{R}={c}^{R}-{c}_{0}$ and $\Delta {c}^{U}={c}^{U}-{c}_{0}$. Note, that $\Delta v/{v}_{0}$ in Eqs. (26) and (27) is the value measured experimentally (Fig. 4a).

### Terahertz-infrared techniques

For the terahertz-infrared studies, plane-parallel plates of about 3 × 3 mm^2^ area were cut with the *c*-axis in the plane of the sample. Such orientation allows to measure polarization-dependent dielectric response for the two principle polarizations of the ${\boldsymbol{{\rm E}}}$-vector of the probing radiation - parallel and perpendicular to the $c$-axis. The measurements were performed in Laboratory of Terahertz Spectroscopy, Moscow Institute of Physics and Technology, Russia and in 1. Physical Institute, University of Stuttgart, Germany. Two types of spectrometers were used: Terahertz time-domain spectrometer (Teraview TPS 3000 TDS) and Fourier-Transform infrared spectrometer (Bruker Vertex 80 FTIR spectrometer with Hyperion 2000 microscope). The measurements were performed in the temperature range $5\mbox{--}300\,{\rm{K}}$ and at frequencies $0.3\mbox{--}240\,{\rm{THz}}$ ($10\mbox{--}8000\,{{\rm{cm}}}^{-1}$). The obtained spectra were processed using Lorentzian and Debye expressions to simulate the resonance and relaxational spectra:

28 $${\varepsilon }^{\ast }(\nu )={\varepsilon }^{{\rm{{\prime} }}}(\nu )+i{\varepsilon }^{{\rm{{\prime} }}{\rm{{\prime} }}}(\nu )=\frac{\Delta {\varepsilon }_{D}}{1+i\frac{\nu }{{\gamma }_{D}}}+\sum _{j}\frac{{f}_{j}}{({\nu }_{j}^{2}-{\nu }^{2})+i\nu {\gamma }_{j}},$$

where ${f}_{j}=\Delta {\varepsilon }_{j}{\nu }_{j}^{2}$ is the oscillator strength of the *j*-th resonance, $\Delta {\varepsilon }_{j}$ is its dielectric contribution, ${\nu }_{j}$ represents the resonance frequency, ${\gamma }_{j}$ the damping factor, $\Delta {\varepsilon }_{D}$ is dielectric contribution of the relaxation to static permittivity, ${\gamma }_{D}$ is the relaxation damping constant.

### Calculation of the primary force constant

The primary force constant ${K}_{E}$ (defined without taking into account of the JTE) that enters Eqs. (12–15) and (18) determines the elastic energy of the JT complex related to symmetrized (in our case of tetragonal type) deformations. In the cluster model used for molecules, it is defined as

29 $${K}_{E}={\omega }_{R}^{2}M,$$

where

30 $$M=4{m}_{O}{m}_{Fe}/(4{m}_{O}+{m}_{Fe}).$$

is the reduced mass of the FeO_4_ complex. Calculation done with ${\omega }_{R}={\omega }_{L1}$ gives ${K}_{E}=15\,{{\rm{J}}/{\rm{m}}}^{2}$. This value provides local deformations of the JT complex ${\rho }_{0}$, Eq. (13), that is unreasonably large since it is comparable to the lattice parameter. The approach based on Eq. (29) is called *ideal problem*. Description of the JT complex in a crystal is much more complicated because the nearest neighbors are coupled within the second coordination sphere as well as to other atoms of the lattice leading to *multimode problem* (see section 3.5 in ^25^). In addition to increase of the number of normal vibrational modes, a coupling with other atomic layers increases the effective mass $M$ of the JT complex. In magnetic crystals, another source of increase of *K*_E_ is the overall rigidity caused by magnetic interaction of the ions. This can be clearly seen if one compares the elastic moduli ${c}_{44}$ of BaFe_12_O_19_ and of CdSe (hexagonal crystal of space group $P{6}_{3}mc$). These two crystals have approximately equal densities, and their doping provides with similar JT complexes. However, the rigidity of the hexaferrite is about three times higher. The force constant of the JT complex plays the same role as the elastic modulus in a crystal: they both define the elastic energy ${E}_{e}$ (or the free energy density $F$) as a function of either displacements (${E}_{e}={K}_{E}({Q}_{2}^{2}+{Q}_{3}^{2})/2$) or deformations ($F=\sum _{i,j}{c}_{ij}{\varepsilon }_{i}{\varepsilon }_{j}$), respectively. Hence, the force constants of similar JT complexes in similar crystals should relate as the corresponding elastic moduli of the crystals. The primary force constant in cadmium selenide was determined as ${K}_{E}^{CdSe}=33.6\,{\rm{J}}/{\rm{m}}$^ 59^. The calculation of the JTE parameters using this value gives a quite reasonable result. In CdSe, the modulus ${c}_{44}^{CdSe}=13.4\times {10}^{9}\,{{\rm{J}}/{\rm{m}}}^{3}$. Using the value of the modulus in BaFe_12_O_19_ ${c}_{44}=37.6\cdot {10}^{9}\,{{\rm{J}}/{\rm{m}}}^{3}$ we may write for the studied hexaferrite:

31 $${K}_{E}=\frac{{c}_{44}}{{c}_{44}^{CdSe}}{K}_{E}^{CdSe}\approx 94\,{{\rm{J}}/{\rm{m}}}^{2}.$$

## Data Availability

The datasets generated during and/or analyzed during the current study are available from corresponding author on reasonable request.

## References

[1] Jahn HA, Teller E (1937). Stability of Polyatomic Molecules in Degenerate Electronic States. I. Orbital Degeneracy. Proc R. Soc. London A.

[2] Bersuker IB (2013). Pseudo-Jahn–Teller Effect—A Two-State Paradigm in Formation, Deformation, and Transformation of Molecular Systems and Solids. Chem. Rev..

[3] Jin YM, Wang YU, Ren Y (2015). Theory and experimental evidence of phonon domain and their role in pre-martensitic phenomena. npj Comput. Mater..

[4] Varignon J, Bristowe NC, Bousquet E, Ghosez P (2015). Coupling and electrical control of structural, orbital and magnetic orders in perovskites. Sci. Rep..

[5] Disseler SM (2015). One Dimensional(1D)-to-2D Crossover of Spin Correlations in the 3D Magnet ZnMn_2_O_4_. Sci. Rep..

[6] Huang HY (2017). Jahn-Teller distortion driven magnetic polarons in magnetite. Nat. Commun..

[7] Gai Z (2014). Chemically induced Jahn–Teller ordering on manganite surfaces. Nat. Commun..

[8] Zhou H (2015). Evolution and control of the phase competition morphology in a manganite film. Nat. Commun..

[9] Puškarova I, Breza M, On M (2015). molecular and electron structures of neutral and charged forms of a dinuclear zinc(II) complex with diphenylamine ligands. Chem. Phys. Lett..

[10] Gali AT (2016). Electron–vibration coupling induced renormalization in the photoemission spectrum of diamondoids. Nat. Commun..

[11] Liu Y (2016). Geometry, Electronic Structure, and Pseudo Jahn-Teller Effect in Tetrasilacyclobutadiene Analogues. Sci. Rep..

[12] Mizuhashi S (1977). Out-of-plane distortion of the iron in ferrohemoglobin due to pseudo-Jahn-Teller effect. J. Theor. Biol..

[13] Bacci M (1980). Jahn-Teller effect in biomolecules. Biophys. Chem..

[14] Wang Y, Liu Y, Zheng X (2018). Pseudo Jahn-Teller origin tracking for symmetry breaking in halogenabenzene: How can a bird fly?. Int. J. Quantum Chem..

[15] Mizuhashi S (1990). Partial Quenching of Off-Diagonal Racah Parameters and Diminishing of Diagonal Elements Due to Dynamical Jahn-Teller Effect T1(dN) and T2(dN) × ε. J. Phys. Soc. Jpn..

[16] Shepidchenko A, Sanyal B, Klintenberg M, Mirbt S (2015). Small hole polaron in CdTe: Cd-vacancy revisited. Sci.Rep..

[17] Butykai A (2017). Characteristics of ferroelectric-ferroelastic domains in Néel-type skyrmion host GaV4S8. Sci. Rep..

[18] Ye H (2017). Excitation Dependent Phosphorous Property and New Model of the Structured Green Luminescence in ZnO. Sci. Rep..

[19] Zhang L (2018). Strong-correlated behavior of 4f electrons and 4f5d hybridization in PrO_2_. Sci. Rep..

[20] Caddeo F, Loche D, Maria F, Casula MF, Corrias A (2018). Evidence of a cubic iron sub-lattice in t-CuFe_2_O_4_ demonstrated by X-ray Absorption Fine Structure. Sci. Rep..

[21] Kozlenko, D. P. et al. Magnetic and electronic properties of magnetite across the high pressure anomaly. *Sci. Rep*. **9**, 4464 (20191). 10.1038/s41598-019-41184-3 PMC6418096 30872759

[22] Klupp G (2012). Dynamic Jahn–Teller effect in the parent insulating state of the molecular superconductor Cs_3_C_60_. Nat. Commun..

[23] Bristowe NC, Varignon J, Fontaine D, Bousquet E, Ghosez P (2015). Ferromagnetism induced by entangled charge and orbital orderings in ferroelectric titanate perovskites. Nat. Commun..

[24] Kajimoto R (2018). Elastic and dynamical structural properties of La and Mn-doped SrTiO_3_ studied by neutron scattering and their relation with thermal conductivities. Sci. Rep..

[25] Bersuker, I. B. *The Jahn-Teller Effec*t (Cambridge University Press, Cambridge, 2006).

[26] Merten S, Shapoval O, Damaschke D, Samwer K, Moshnyaga V (2019). Magnetic-Field-Induced Suppression of Jahn-Teller Phonon Bands in (La._0.6_Pr_0.4_)_0.7_Ca_0.3_MnO_3_: the Mechanism of Colossal Magnetoresistance shown by Raman Spectroscopy. Sci. Rep..

[27] Ivanov PA (2016). High-precision force sensing using a single trapped ion. Sci. Rep..

[28] Seo H (2016). Design of defect spins in piezoelectric aluminum nitride for solid-state hybrid quantum technologies. Sci. Rep..

[29] Tsukerblat B, Palii A, Clemente-Juan JM, Coronado E (2017). Jahn-Teller effect in molecular electronics: quantum cellular automata. J. Phys.: Conf. Ser..

[30] Obradors X, Collomb A, Pernet M, Samaras D, Joubert JC (1985). X-ray analysis of the structural and dynamic properties of hexagonal ferrite at room temperature. J. Solid State Chem..

[31] Sturge M.D. The Jahn-Teller Effect in Solids, in Solid State Physics, edited by Seitz, F., Turnbull, D. & Ehrenreich, H., **20**, 92–211 (Academic Press, New York, London, 1967).

[32] Pappalardo R, Wood DL, Linares RC (1961). Optical absorption study of Co-doped oxides systems. II. J. Chem. Phys..

[33] McClure DS (1962). Optical Spectra of Transition-Metal Ions in Corundum. J. Chem. Phys..

[34] Vallin JT, Slack GA, Roberts S, Hughes AE (1970). Infrared absorption in some II-VI compounds doped with Cr. Phys. Rev. B..

[35] Vallin JT, Watkins GD (1974). EPR of Cr^2+^ in II-VI lattices. Phys. Rev. B.

[36] Abou-Ghantous, M., Bates, C. A., Fletcher, J. R. & Jaussaud, P. C. Further studies of the Ni^3+^: Al_2_O_3_ Jahn-Teller system. *J. Phys. C: Solid State Phys.* **8**, 3641–3652 (1975).

[37] Gyorgy EM, Sturge MD, Fraser DB, LeCraw RC (1965). Observation of the Jahn-Teller tunneling by acoustic loss. Phys. Rev. Lett..

[38] Tokumoto H, Ishiguro T (1979). Ultrasonic study of dynamic behavior of Jahn-Teller distorted Cr^2+^ centers in GaAs. J. Phys. Soc. Jpn..

[39] Gudkov VV, Lonchakov AT, Sokolov VI, Zhevstovskikh IV (2006). Relaxation in ZnSe:Cr^2+^ investigated with longitudinal ultrasonic waves. Phys. Rev. B.

[40] Gudkov VV, Lonchakov AT, Sokolov VI, Zhevstovskikh IV, Surikov VT (2008). Ultrasonic investigation of ZnSe:V^2+^ and ZnSe:Mn^2+^: Lattice softening and low-temperature relaxation in crystals with orbitally degenerate states. Phys. Rev. B.

[41] Averkiev NS (2017). Magnetic field induced tunneling and relaxation between orthogonal configurations in solids and molecular systems. Phys. Rev. B.

[42] Averkiev, N. S. et al. Jahn-Teller effect problems via ultrasonic experiments. Application to the impurity crystal CdSe: Cr. *J. Phys.: Conf. Ser*. 1148 (2018).

[43] Averkiev, N. S. *et al*. The Jahn-Teller Effect in Elastic Moduli of Cubic Crystals: General Theory and Application to Strontium Fluorite Doped with Chromium Ions. In Fluorites, ed. M. van Asten (Nova Science Publishes, Inc., New York, pp.111–159, 2019).

[44] Gudkov, V. V. & Bersuker, I. B. Experimental Evaluation of the Jahn-Teller Effect Parameters by Means of Ultrasonic Measurements. Application to Impurity Centers in Crystals in Vibronic Interaction and the Jahn-Teller Effect eds. Atanasov, M., Daul, C., Treggenna-Piggot, P. L. (Heidelberg: Springer, pp 143–161, 2012).

[45] Ishiwata S, Taguchi Y, Murakawa H, Onose Y, Tokura Y (2008). Low-magnetic-field control of electric polarization vector in a helimagnet. Science.

[46] Kitagawa Y (2010). Low-field magnetoelectric effect at room temperature. Nat. Mater..

[47] Tokunaga Y (2010). Multiferroic M-Type Hexaferrites with a Room-Temperature Conical State and Magnetically Controllable Spin Helicity. Phys. Rev. Lett..

[48] Pullar RC (2012). Hexagonal ferrites: A review of synthesis, properties and applications of hexaferrite ceramics. Prog. Mater. Sci..

[49] Kimura T (2012). Magnetoelectric hexaferrites. Annu. Rev. Condens. Matter Phys..

[50] Mikheykin AS (2014). Lattice anharmonicity and polar soft mode in ferromagnetic M-type hexaferrite BaFe_12_O_19_ single crystal. Eur. Phys. J. B.

[51] Alyabyeva LN (2019). Influence of chemical substitution on broadband dielectric response of barium-lead M-type hexaferrite. New J. Phys..

[52] Atuchin VV (2016). Flux Crystal Growth and the Electronic Structure of BaFe_12_O_19_ Hexaferrite. J. Phys. Chem. C.

[53] Rensen JG, van Wieringen JS (1969). Anisotropic Mossbauer fraction and crystal structure of BaFe_12_O_19_. Solid State Commun..

[54] Collomb A, Wolfers P, Obradors X (1986). Neutrom diffraction studies of some hexaferrites: BaFe_12_O_19_, BaMg_2__W and BaCo_2__W. J. Magn. Magn. Mater..

[55] Vinnik DA (2014). Ti-Substituted BaFe_12_O_19_ single crystal growth and characterization. Cryst. Growth Des..

[56] Truell, R., Elbaum, C. & Chick, B. B. *Ultrasonic Methods in Solid State Physics*. (Academic Press, New York and London, 1969).

[57] Averkiev NS (2017). Elastic moduli in cadmium selenide doped with chromium. Journal of Applied Mathematics and Physics.

[58] Tucker, J. W. & Rampton, V. W. *Microwave ultrasonics in solid state physics*. (North-Holland Publishing Company, Amsterdam, 1972).

[59] Averkiev, N. S. *et al*. Ultrasonic Determination of the Jahn–Teller Effect Parameters in Impurity-Containing Crystals. *J. Exp. Theor. Phys.* **129**, 72–80 (2019).

[60] Kawai Y, Barbers VAM, Šimša Z, Dalderop JHJ (1999). Ultrasonic attenuation in BaTiFe_11_O_19_ single crystal. J. Magn. Magn. Mater..

[61] Alyabyeva, L. *et al.* Terahertz-infrared spectroscopy of Ti^4+^-doped M-type barium hexaferrite. *J. Alloys Compd*. **820**, 153398 (2020).

[62] Marino-Castellanos PA, Anglada-Rivera J, Cruz-Fuentes A, Lora-Serrano R (2004). Magnetic and microstructural properties of the Ti^4+^-doped Barium hexaferrite. J. Magn. Magn. Mater..

[63] Cabinas MV, Gonsalez-Calbet JM, Rodriguez-Carvajal J, Vallet-Regi M (1994). The solid solution BaFe12-2xCoxTix O19 (0 ≤ x ≤ 6): Cationic Distribution by Neutron Diffraction. J. Solid State Chem..

[64] Gudkov, V. V. Ultrasonic Consequences of the Jahn–Teller Effect in The Jahn–Teller Effect eds. H. Koppel, H., Yarkony, D. R. & and Barentzen, H. (Springer, New York, 2009).

[65] Zener, C. *Elasticity and Anelasticity of Metals*. (University of Chicago Press, Chicago, 1948).

[66] Gudkov VV (2018). Magnetoacoustic relaxation by Cr^2+^ Jahn-Teller centers revealed from elastic moduli. Phys. Status Solidi A.

[67] Amer MA, Meaz TM, Attalah SS, Ghoneim AI (2015). Structural and magnetic studies of Ti^4+^ substituted M-type BaFe_12_O_19_ hexa-nanoferrites. Materials Science in Semiconductor Processing.

[68] Zherlitsyn S (2014). Spin-lattice effects in selected antiferromagnetic materials. Low Temp. Phys..

[69] Wolf B (2001). New experimental techniques for pulsed magnetic fields - ESP and ultrasonic. Physica B.

